# Development and validation of a nomogram for prediction of the risk of positive hidden blood loss in the perioperative period of single-level thoracolumbar burst fracture

**DOI:** 10.1186/s13018-021-02699-6

**Published:** 2021-09-15

**Authors:** Haosheng Wang, Tingting Fan, Zhi-Ri Tang, Wenle Li, Linjing Liu, Qiang Lin

**Affiliations:** 1 Department of Orthopedics, Baoji City Hospital of Traditional Chinese Medicine, 43 Baofu Road, Baoji, 721000 Shaanxi Province People’s Republic of China; 2 Department of Endocrinology, Baoji City Hospital of Traditional Chinese Medicine, Baoji, Shaanxi Province People’s Republic of China; 3 Department of Computer Science, City University of Hong Kong, Hong Kong, People’s Republic of China; 4 Department of Orthopedics, Xianyang Central Hospital, Xianyang, People’s Republic of China; 5 Clinical Medical Research Center, Xianyang Central Hospital, Xianyang, People’s Republic of China

**Keywords:** Hidden blood loss, Blood loss, Thoracolumbar burst fracture, Risk factors, Nomogram

## Abstract

**Background:**

This study aimed to develop and validate an individualized nomogram to predict the risk of positive hidden blood loss (HBL) in patients with single-level thoracolumbar burst fracture (TBF) during the perioperative period.

**Methods:**

We conducted a retrospective investigation including 150 consecutive patients with TBL, and the corresponding patient data was extracted from March 2013 to March 2019. The independent risk factors for positive HBL were screened using univariate and multivariate logistic regression analyses. According to published literature and clinical experience, a series of variables were selected to develop a nomogram prediction model for positive HBL. The area under the receiver operating characteristic curves (AUC), C-index, calibration plot, and decision curve analysis (DCA) were used to evaluate the performance of the prediction model. Bootstrapping validation was performed to evaluate the performance of the model.

**Results:**

Among the 150 consecutive patients, 62 patients were positive for HBL (38.0%). The multivariate logistic regression analysis showed that the six risk factors of age, length of surgical incision, duration of operation, percentage of vertebral height restoration (P_1_%), preoperative total cholesterol, and preoperative fibrinogen were independent risk factors of positive HBL. The C-index was 0.831 (95% CI 0.740–0.889) and 0.845 in bootstrapping validation, respectively. The calibration curve showed that the predicted probability of the model was consistent with the actual probability. Decision curve analysis (DCA) showed that the nomogram had clinical utility.

**Conclusion:**

Overall, we explored the relationship between the positive HBL requirement and predictors. The individualized prediction model for patients with single-level TBF can accurately assess the risk of positive HBL and facilitate clinical decision making. However, external validation will be needed in the future.

**Supplementary Information:**

The online version contains supplementary material available at 10.1186/s13018-021-02699-6.

## Introduction

Thoracolumbar fractures are commonly encountered in clinical practice, and fractures of the burst type account for 25 to 58% [1–3] of all thoracolumbar spinal fractures. Thoracolumbar burst fractures (TBF) shorten people’s lives and cause impairment in the quality of life [2–4]. Surgical intervention is often needed to alleviate pain, rebuild spinal stability, restore function, and relieve spinal cord compression. Meanwhile, surgical intervention can also facilitate nursing care and reduce prolonged bed rest complications [5, 6].

Remarkably, intraoperative blood loss is a prevalent issue that can be encountered in the surgical treatment of thoracolumbar burst fractures [7, 8]. Nevertheless, patients with thoracolumbar burst fractures occasionally require a transfusion despite what appears to be a limited intraoperative blood loss. This may be due to undetectable blood loss, called hidden blood loss (HBL). The concept of HBL was first described in 2000 by Sehat et al. [9] who found that HBL accounted for 26% and 49% of total blood loss after total knee and hip replacement, respectively. Subsequently, many studies about HBL after orthopedic surgery have been widely concerned by many scholars. There have been several reports on HBL in spine surgery, including anterior cervical fusion, percutaneous kyphoplasty, posterior lumbar decompression and fusion (PLDF), single-level open or minimally invasive transforaminal interbody fusion (MIS-TLIF) [10–13]. Nevertheless, few reports associated with HBL in thoracolumbar burst fractures are available.

The HBL during the perioperative period is usually ignored, but it is important for the prognosis and outcome. Despite satisfactory management of blood loss during the perioperative period, it is puzzling that patient with thoracolumbar burst fractures often have lower postoperative HB levels than expected. We therefore deduced that it might be due to HBL. Unfortunately, however, few studies have been conducted to investigate the HBL in patients with thoracolumbar burst fractures during the perioperative period. In this study, we aimed to explore the risk factors related to HBL in patients with single-level TBF and develop an accurate individualized risk of HBL predictive tool based on routine parameters.

## Materials and methods

The study protocol was approved by the Baoji City Hospital of Traditional Chinese Medicine (Baoji, China) and the Research Ethics review board (NO. 2021GYKJD5N). Due to the retrospective nature of the study, a waiver of informed consent was obtained. From March 2013 to March 2019, patients with symptomatic single-level TBF were obtained and analyzed. The inclusion criteria for the study were as follows: (1) age of ≥18; (2) burst fracture (AO classification); (3) fracture level: T11-L2; (4) Thoracolumbar Injury Classification and Severity Score (TLICS) ≥ 5 [14, 15]; and (5) surgical approach: posterior instrumentation. The exclusion criteria were as follows: (1) organ with dysfunction such as liver or kidney, abnormal bleeding, or abnormal coagulation function; (2) history of surgery for spinal disorder; (3) cerebrospinal fluid leakage (CSF fistula) or spontaneous; (4) intraoperative and postoperative use of hemostatic drugs; and (5) patients with incomplete medical records. HBL ≥ 470ml was defined as an HBL-positive group, according to the definition of HBL, calculated by gross equation, and HBL < 470ml was an HBL negative group [16].

### Data collections

We retrospectively collected the patients’ data, including age, sex, body mass index (BMI), underlying diseases (hypertension, diabetes, chronic obstructive pulmonary disease (COPD)), history of smoking, history of alcohol, history of blood transfusion, and chronic steroid use. The preoperative visual analogue score (VAS), Japanese Orthopaedic Association (JOA) scores, and 36-Item Short Form Health Survey (SF-36) were recorded.

Surgery-related parameters including duration from admission to surgery, length of surgical incision, levels of fusion, and duration of operation were obtained from an electronic medical record system. The parameters relevant to the perioperative fluid management strategy, including intraoperative infusion of crystalloids, intraoperative infusion of colloids, autologous blood transfusion, and allogeneic blood transfusion, were recorded. Meanwhile, routine preoperative laboratory test data, including hematologic tests, blood chemistry, coagulation tests, and liver function tests, was obtained from the clinical laboratory database of the Baoji City Hospital of Traditional Chinese Medicine. In addition, to assess the influence of fractured vertebral height on hidden blood loss, we calculated percentages of vertebral height restoration (P_1_%) and vertebral height loss (P_2_%) by measuring preoperative and postoperative X-ray parameters [7]. The formulae for P_1_% and P_2_% calculation are as follows:

$$ \mathrm{P}1\%=\frac{e-a}{d}\times 100\% $$

$$ \mathrm{P}2\%=\frac{d-a}{d}\times 100\% $$

$$ d=\frac{b+c}{2} $$

**Fig. 1:**
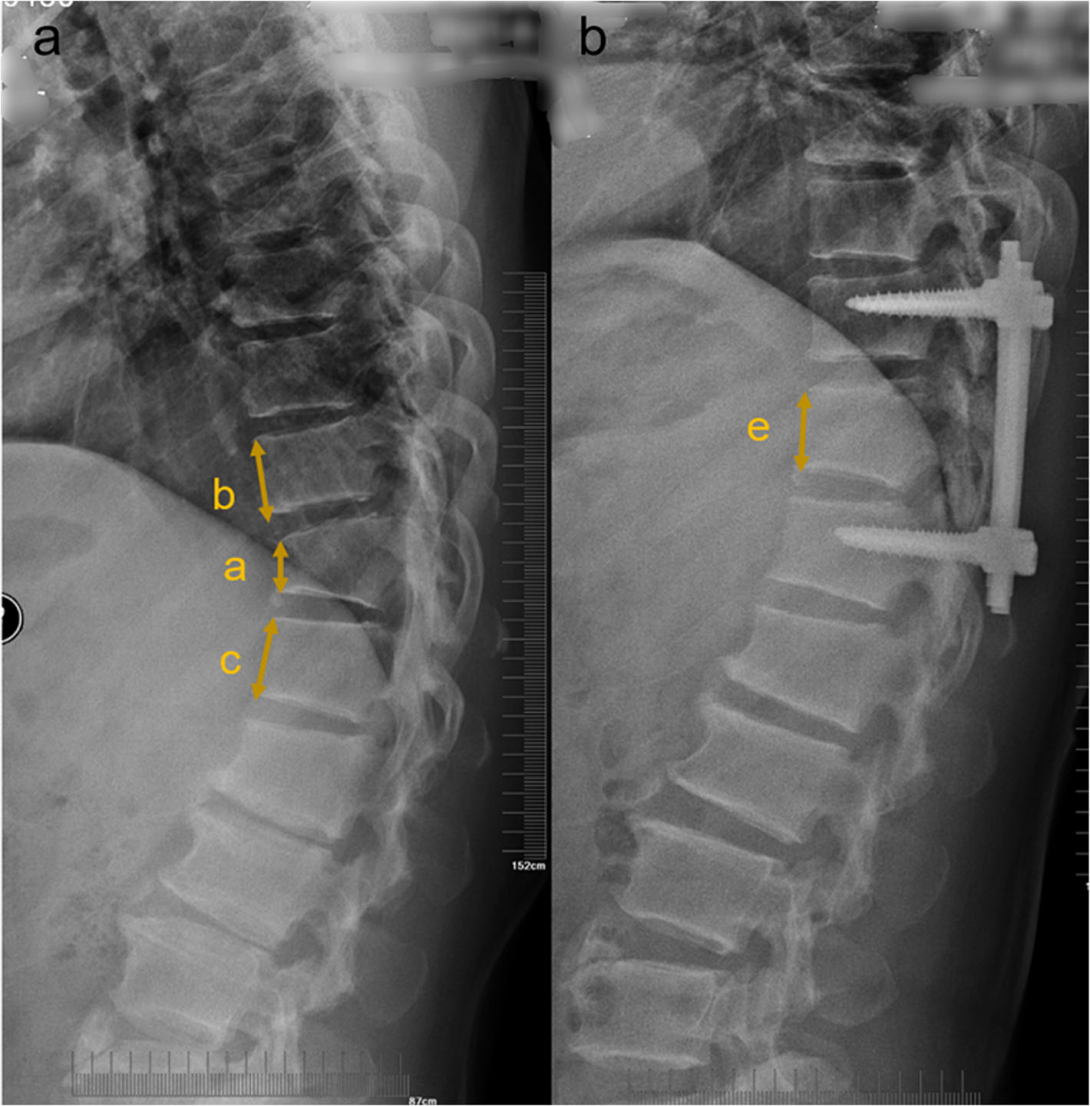
Radiographic measurement of fractured vertebral body. **a** Preoperative lateral radiograph; *a* is the height of the fractured vertebra; *b* is the upper anterior vertebral height adjacent to the fractured vertebra, and *c* is the lower anterior vertebral height adjacent to the fractured vertebra. **b** Postoperative lateral radiograph; *e* is the postoperative vertebral body height. *Note*: *d* is the predicted height of each fractured vertebra which is calculated according to the average height of the two adjacent vertebrae (*b* and *c*)

Here, *a* is the height of the fractured vertebra, *b* is the upper anterior vertebral height adjacent to the fractured vertebra, *c* is the lower anterior vertebral height adjacent to the fractured vertebra, *d* is the predicted height of each fractured vertebra which is calculated according to the average height of the two adjacent vertebrae (*b* and *c*), and *e* is the postoperative vertebral body height. This parameter measurement method is shown in Fig. 1.

### Management and calculation of HBL

According to the formula by Nader et al. [17], patient’s blood volume (PBV) was calculated as follows:

$$ \mathrm{PBV}=\mathrm{k}1\times \mathrm{height}\ \left(\mathrm{m}\right)+\mathrm{k}2\times \mathrm{weight}\ \left(\mathrm{kg}\right)+\mathrm{k}3 $$

Here, for male, k1 = 0.3669, k2 = 0.03219, and k3 = 0.6041; for female, k1 = 0.3561, k2 = 0.03308, and k3 = 0.1833.

According to the gross formula, total blood loss (TBL) was calculated as follows:

$$ \mathrm{TBL}=\mathrm{PBV}\times \left({\mathrm{Hct}}_{\mathrm{pre}}-{\mathrm{Hct}}_{\mathrm{post}}\right)/{\mathrm{Hct}}_{\mathrm{ave}} $$

where Hct_pre_ was defined as the Hct on preoperative day 1, Hct_post_ was defined as the Hct on postoperative day 3, and Hct_ave_ was defined was the average of Hct_pre_ and Hct_post_. Subsequently, intraoperative blood loss was determined as follows:

$$ \mathrm{Intraoperative}\ \mathrm{blood}\ \mathrm{loss}=\mathrm{estimating}\ \mathrm{the}\ \mathrm{volume}\ \mathrm{of}\ \mathrm{blood}\ \mathrm{in}\ \mathrm{the}\ \mathrm{suction}\ \mathrm{container}+\mathrm{weighing}\ \mathrm{blood}-\mathrm{soaked}\ \mathrm{gauze} $$

$$ \mathrm{Visible}\ \mathrm{blood}\ \mathrm{loss}\ \left(\mathrm{VBL}\right)=\mathrm{intraoperative}\ \mathrm{blood}\ \mathrm{loss}+\mathrm{postoperative}\ \mathrm{drainage} $$

Therefore, HBL was defined as follows:

$$ \mathrm{HBL}=\mathrm{TBL}-\mathrm{VBL} $$

when there was perioperative blood transfusion, HBL could be defined as follows:

HBL = TBL – VBL + autologous blood transfusion + allogeneic blood transfusion

### Statistical analysis

Continuous data is presented as the means ± standard deviations. Categorical variables were grouped and compared using the *χ*^2^ test or Fisher’s exact test. Continuous variables were compared using Student’s *t*-test. Risk factor analysis was performed using univariate and multivariate logistic regression analyses. In order to facilitate the establishment of the prediction model, cut-off values for continuous variables were determined by the receiver operating characteristic (ROC) curve (Figure S1). Variables showing statistical significance in the univariate analysis were included in the multivariate logistic regression analysis, and the forward stepwise method was used to select the variables that were eventually included in the model. According to the results of the regression coefficients of independent variables, an individual nomogram prediction model for HBL was established. The performance of the model was assessed in terms of discrimination and calibration. The discrimination of our prediction model is usually evaluated by calculating the area under the curve (AUC) of the ROC curve. Typically, the AUC values of the models were between 0 and 1. A prediction model with an AUC in the range of 0.5–0.75 was considered acceptable, and AUC > 0.75 indicated that the models possessed excellent discriminative power. The calibration of the nomogram was evaluated with a calibration curve. The Hosmer–Lemeshow test was conducted to assess the goodness-of-fit of the nomogram. A relatively corrected C-index (1000 bootstrap resamples) of the nomogram was also determined in this dataset. Furthermore, the decision curve analysis (DCA) was performed to evaluate the net benefit of the nomogram to the decision. All the analyses were performed by using IBM SPSS 23.0 (SPSS Inc) and R software version 4.0.2 (http://www.r-project.org) with the “rms,” “SimDesign,” and “AICcmodavg” packages [18–20].

## Results

In this study, a total of 178 consecutive cases were collected. According to the inclusion and exclusion criteria, 5 intraoperative dural ruptures, 10 postoperative tranexamic acid use, 2 intraoperative thrombin drug use, and 11 missing laboratory parameters about Hct_post_, 28 patients were excluded. Finally, 150 cases were included, 99 (66.0%) males and 51 (34.0%) females, mean (38.1 ± 10.3) years old. The TBL was 1134.5±128.3 ml, the VBL was 269.1±82.5 ml, and the HBL was 491.3±104.5 ml (50.3 % of TBL). Among the 150 cases, 93 cases were negative HBL, i.e., HBL (−) group; 57 cases were positive HBL, i.e., HBL (+) group.

Among the HBL (−) and HBL (+) group, there were statistically significant differences in age (*p* < 0.001), hypertension (*p* < 0.001), history of smoking (*p* < 0.001), diabetes (*p* = 0.017), duration from admission to surgery (*p* = 0.021), length of surgical incision (*p* < 0.001), duration of operation (*p* < 0.001), P_1_% (*p* < 0.001), P_2_% (*p* < 0.001), preoperative total cholesterol (*p* < 0.001), preoperative triglyceride (*p* < 0.001), preoperative Hb (*p* = 0.017), preoperative PT (*p* < 0.001), preoperative Fib (*p* = 0.038), and preoperative APTT (*p <* 0.001). There were no significant differences in remaining parameters between the HBL (−) and HBL (+) group. The detailed results of the parameters are shown in Table 1.

**Table 1:** Comparison of variables between the positive HBL group and negative HBL group

	Total	Negative HBL group	Positive HBL group	*P* value
Number of patients	150	93	57	
Age (year)	38.1 (10.3)	34.3 (9.5)	49.2 (8.1)	<0.001
Sex (%)				
Female	99 (66.0)	60 (64.5)	39 (68.4)	0.711
Male	51 (34.0)	33 (35.5)	18 (31.6)	
BMI (%)				
< 25	77 (51.3)	53 (57.0)	24 (42.1)	0.065
≧25	73 (48.7)	40 (43.0)	33 (57.9)	
Hypertension (%)				
No	56(37.3)	45 (48.4)	11 (19.3)	<0.001
Yes	94 (62.7)	50 (51.6)	46 (80.7)	
Diabetes (%)				
No	63 (42.0)	53 (57.0)	10 (17.5)	0.017
Yes	87 (58.0)	40 (43.0)	47 (82.5)	
History of smoking (%)				
No	77 (51.3)	61 (65.6)	16 (28.1)	<0.001
Yes	73 (48.7)	32 (34.4)	41 (71.9)	
History of alcohol (%)				
No	68 (42.5)	40 (38.8)	28 (49.1)	0.165
Yes	92 (57.5)	63 (61.2)	29 (50.9)	
Chronic steroid use (%)				
No	133 (88.7)	80 (86.0)	53 (93.0)	0.321
Yes	17 (11.3)	13 (14.0)	4 (7.00)	
COPD (%)				
No	145 (96.7)	90 (96.8)	55 (96.5)	0.965
Yes	5 (3.30)	3 (3.20)	2 (3.50)	
History of blood transfusion (%)				
No	149 (99.3)	92 (98.9)	57 (100.0)	0.421
Yes	1 (0.7)	1 (1.10)	0 (0.00)	
Preoperative VAS	5.9 [4.9, 7.1]	6.8 [4.8, 7.0]	5.8 [4.9, 7.0]	0.265
Preoperative JOA	12.7 [10.0, 16.5]	13.1 [10.0, 16.0]	13.2 [10.8, 15.5]	0.228
Preoperative SF-36	70.6 (5.4)	71.3 (6.0)	69.9 (6.8)	0.65
Duration from admission to surgery (day)	0.6 (0.5)	0.48 (0.5)	0.72 (0.3)	0.021
Length of surgical incision (cm)	14.0 (1.6)	12.9 (1.1)	15.8 (0.9)	<0.001
Duration of operation (min)	164.6 (18.4)	157.0 (13.8)	178.2(15.5)	<0.001
P_1_%	38.3 [36.7, 44.1]	37.2 [36.0, 36.4]	46.0 [41.4, 48.1]	<0.001
P_2_%	29.7 [27.0, 31.8]	30.9 [26.9, 34.7]	28.5 [26.9, 30.9]	0.011
Level				
T11	64 (43.0)	43 (46.1)	22 (37.9)	0.637
T12	63 (41.9)	38 (40.8)	25 (43.6)	
L1	23 (15.2)	12 (13.1)	11 (18.5)	
Levels of fusion (%)				
T9-L1	73 (48.7)	50 (53.8)	23 (40.4)	0.4408
T10-L2	61 (40.7)	36 (38.7)	25 (43.9)	
T11-L3	16 (10.7)	7 (7.50)	9 (15.8)	
Intraoperative infusion of crystalloids (ml)	1569.0 [1317.0, 1862.3]	1561.1 [1359.5, 1856.2]	1538.1 [1239.2, 1889.9]	0.706
Intraoperative infusion of colloids (ml)	812.4 [627.0, 995.5]	809.6 [618.0, 1011.3]	815.1 [670.2, 939.5]	0.876
Autologous blood transfusion (%)				
No	120 (80.0)	77 (82.8)	43 (75.4)	0.470
Yes	30 (20.0)	16 (17.2)	14 (24.6)	
Allogeneic blood transfusion (%)				
No	115 (76.7)	75 (80.6)	40 (70.2)	0.775
Yes	35 (23.3)	18 (19.4)	17 (29.8)	
Preoperative total cholesterol (mmol/L)	4.7 [3.3, 6.4]	3.7 [3.2, 5.0]	6.8 [5.2, 7.0]	<0.001
Preoperative triglyceride (mmol/L)	2.1 [1.4, 3.5]	1.7 [1.0, 2.3]	3.8 [3.2, 4.9]	<0.001
Preoperative Hct (%)	40.9 (3.5)	40.3 (3.9)	42.6(4.4)	0.405
Preoperative Hb (g/L)	122.8 (13.4)	124.2 (15.2)	119.8 (10.2)	0.017
Preoperative ALB (g/L)	39.7 (4.8)	39.2 (4.0)	40.6 (4.3)	0.040
Preoperative PT (s)	10.2 (0.6)	10.1 (0.4)	10.9 (0.5)	<0.001
Preoperative APTT (s)	33.4 (2.7)	35.7 (2.8)	32.9 (2.8)	<0.001
Preoperative Fib (mg/dL)	4.7 (0.9)	4.9 (1.0)	4.6 (0.7)	0.038

*Abbreviations: BMI* body mass index, *COPD* chronic obstructive pulmonary disease, *JOA* Japanese Orthopaedic Association, *VAS* visual analogue score, *SF-36* 36-Item Short Form Health Survey, *P*_*1*_*%* percentages of vertebral height restoration, *P*_*2*_*%* percentages of vertebral height loss, *Hct* hematocrit, *Hb* hemoglobin, *ALB* albumin, *PT* prothrombin time, *APTT* activated partial thromboplastin time

**Fig. 2:**
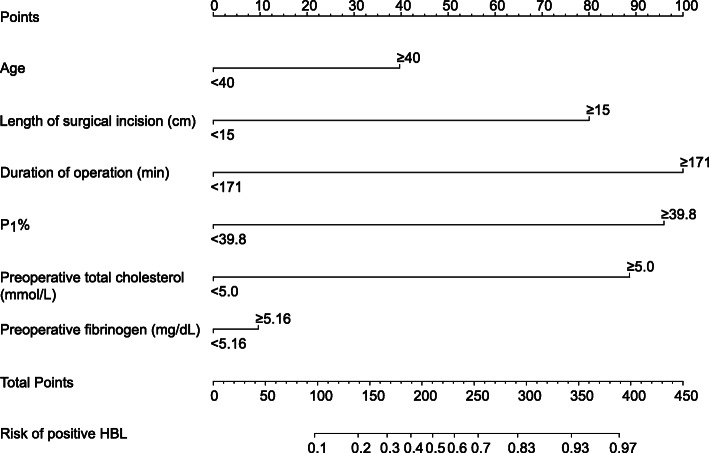
A nomogram to predict the risk of positive HBL. HBL, hidden blood loss; P_1_%, percentages of vertebral height restoration

The univariate logistics analysis showed that statistically significant risk factors were age, hypertension, diabetes, history of smoking, COPD, duration from admission to surgery, length of surgical incision, duration of operation, P_1_%, intraoperative infusion of crystalloids, preoperative total cholesterol, preoperative triglyceride, preoperative ALB, preoperative PT, and preoperative fibrinogen (*P* < 0.05). Statistically significant variables selected from the univariate logistics analysis were imported into the non-conditional binary multivariate logistic regression. The six factors of age, duration from admission to surgery, duration of operation, P_1_%, preoperative total cholesterol, and preoperative fibrinogen were independent risk factors of positive HBL (Table 2) (*P*<0.05). Next, we developed a nomogram based on the result of logistic regression analysis (Fig. 2).

**Table 2:** Univariate and multivariate logistic regression model analyses of positive HBL in this study

	Univariate analysis		Multivariate analysis	
	OR (95% CI)	*P* value	OR (95% CI)	*P* value
Age (year)	4.055 (2.417–6.805)	<0.001	3.056 (1.956–4.965)	0.012
Sex (%)	1.099 (0.663–1.822)	0.715	NA	
BMI (%)	1.668 (1.021–2.726)	0.041	NA	
Hypertension (%)	2.242 (1.351–3.721)	0.002	NA	
Diabetes (%)	2.584 (1.548–4.316)	<0.001	NA	
History of smoking (%)	2.735 (1.651–4.531)	<0.001	NA	
History of alcohol (%)	0.848 (0.520–1.383)	0.509	NA	
Chronic steroid use (%)	1.391 (0.800–2.419)	0.242	NA	
COPD (%)	2.589 (1.273–5.265)	0.009	NA	
History of blood transfusion (%)	1.169 (0.611–2.237)	0.637	NA	
Preoperative VAS	1.444 (0.832–2.506)	0.192	NA	
Preoperative JOA score	1.096 (0.635–1.893)	0.741	NA	
Preoperative SF 36	0.665 (0.395–1.119)	0.125	NA	
Duration from admission to surgery (day)	2.344 (1.418–3.874)	<0.001	1.985 (1.085–2.956)	0.032
Length of surgical incision (cm)	7.352 (4.235–12.761)	<0.001	NA	
Duration of operation (h)	8.906 (5.078–15.621)	<0.001	4.571 (1.787–11.692)	0.002
P_1_%	16.369 (8.826–30.356)	<0.001	3.400 (1.302–8.878)	0.012
P_2_%	0.667 (0.402–1.109)	0.119	NA	
Level	0.817 (0.498–1.450)	0.952	NA	
Levels of fusion (%)	1.639 (1.114–2.412)	0.012	NA	
Intraoperative infusion of crystalloids (ml)	0.569 (0.341–0.949)	0.031	NA	
Intraoperative infusion of colloids (ml)	0.765 (0.469–1.248)	0.283	NA	
Autologous blood transfusion (%)	1.019 (0.601–1.730)	0.943	NA	
Allogeneic blood transfusion (%)	1.771 (1.012–3.097)	0.045	NA	
Preoperative total cholesterol (mmol/L)	9.384 (5.287–16.655)	<0.001	7.161 (2.523–20.322)	<0.001
Preoperative triglyceride (mmol/L)	5.196 (3.059–8.828)	<0.001	NA	
Preoperative Hct (%)	1.576 (0.965–2.576)	0.069	NA	
Preoperative Hb (g/L)	0.713 (0.436–1.167)	0.178	NA	
Preoperative ALB (g/L)	4.478 (2.519–7.959)	<0.001	NA	
Preoperative PT (s)	13.872 (7.632–25.214)	0.089	NA	
Preoperative APTT (s)	0.700 (0.424–1.156)	0.164	NA	
Preoperative Fib (mg/dL)	0.525 (0.317–0.868)	0.012	0.378 (0.179–0.598)	0.029

*Abbreviations: BMI* body mass index, *COPD* chronic obstructive pulmonary disease, *JOA* Japanese Orthopaedic Association, *VAS* visual analogue score, *SF-36* 36-Item Short Form Health Survey, *P*_*1*_*%* percentages of vertebral height restoration, *P*_*2*_*%* percentages of vertebral height loss, *Hct* hematocrit, *Hb* hemoglobin, *ALB* albumin, *PT* prothrombin time, *APTT* activated partial thromboplastin time, *OR* odds ratio, *CI* confidence interval, *NA* not available

**Fig. 3:**
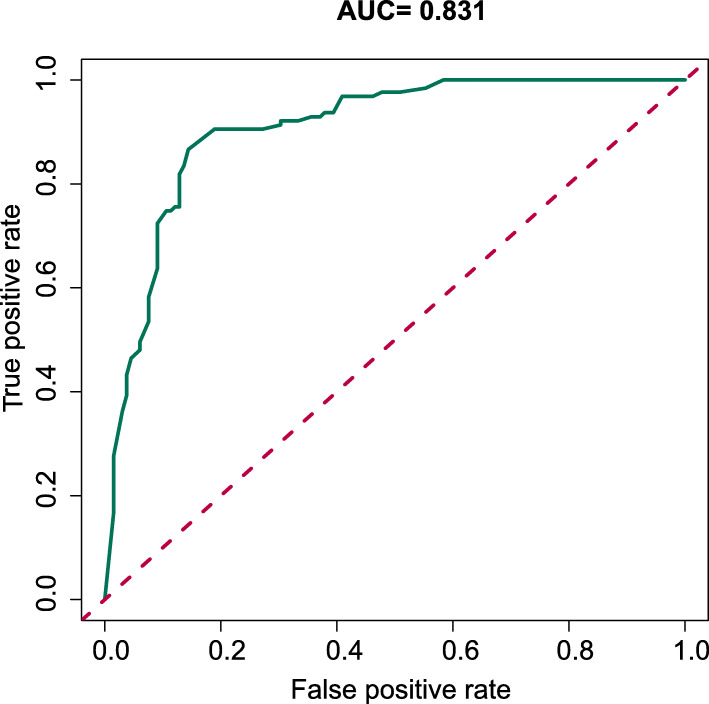
Receiver operating characteristic curve analysis—model validation

**Fig. 4:**
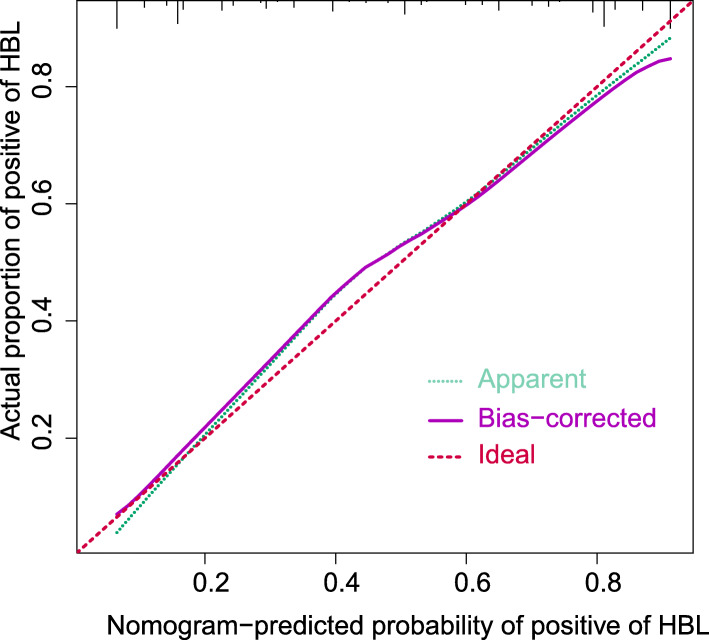
Calibration curves of the nomogram. The lines in the figure represent the apparent value, the bias corrected value, and ideal value. The apparent and the bias corrected values are close to each other, which means the nomogram has a good predictive performance

The area under the curve (AUC) of the receiver operating characteristic (ROC) curve (Fig. 3) of the nomogram was 0.831 (95% CI, 0.740 to 0.889), which indicated that our prediction model showed satisfied discrimination. The calibration curve of the nomogram showed good agreement between the observation cases and prediction cases in this dataset (Fig. 4). Subsequently, the C-index of this model was 0.843 (95% CI 0.749 to 0.905) in this dataset and was identified to be 0.845 via bootstrapping validation (Bootstrap = 1000).

**Fig. 5:**
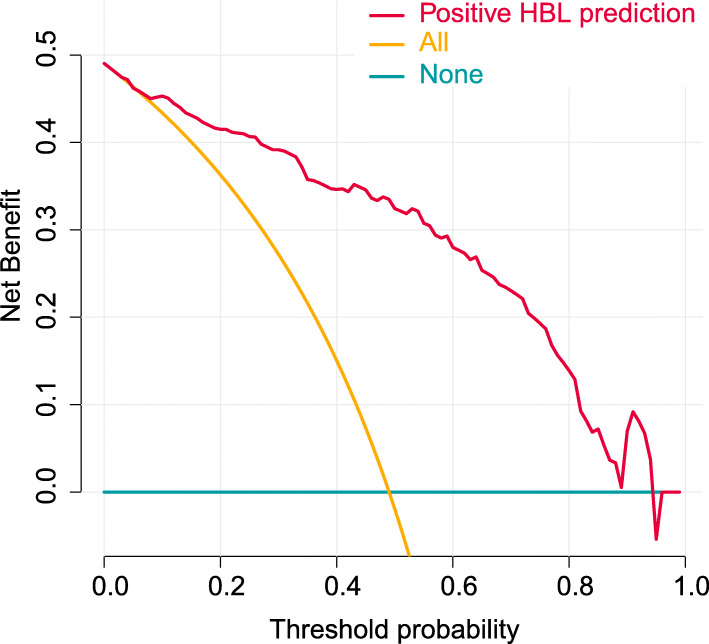
Decision curve analysis for nomogram prediction of risk of positive HBL in the perioperative period of single-level thoracolumbar burst fracture. The *y*-axis shows the net benefit: *x*-axis shows the threshold probability. The blackish green line represents the net benefit of our nomogram. The yellow line indicates the hypothesis that all patients had positive HBL. The blue line represents the hypothesis that no patients had positive HBL

To evaluate the clinical usefulness of the predictive model, a decision analysis (DCA) was performed on the data. The DCA is a novel method that assesses the clinical net benefit of the nomogram. The DCA is demonstrated in Fig. 5. For clinicians and patients, if the threshold was set at 8.1–95.2 % and above, the use of this model to predict the probability of patient transfusion is more beneficial than this scheme.

## Discussion

HBL refers to blood loss that cannot be measured directly in vivo after some of the larger trauma and surgery that is experienced, and this part excludes blood loss that can be quantified directly and postoperative drainage [21]. At present, the mechanism of HBL remains unclear, and some scholars believe that HBL is related to trauma, incomplete intraoperative hemostasis, and postoperative anticoagulant therapy. Another part of scholars believe that it is caused by the accidental opening of the capillary bed due to intraoperative hemolysis, the entrance of a large amount of blood into the body cavity in the perioperative period, and the entrance of marrow fat into the blood circulation. Since Sehat et al. [21] reported that HBL in patients undergoing total hip arthroplasty was 49% of the TBL, spine surgeons are becoming increasingly aware that HBL plays an important role in spine surgery. An increasing number of studies have attempted to investigate the perioperative HBL and its related risk factors in spine surgery. In a prospective study, Smorgick et al. [22] reported that patients undergoing posterior lumbar internal fixation (PLIF) had 600 ml of HBL, which accounts for 42% of the TBL [23]. In another retrospective study, Wen et al. [8] reported that HBL in thoracolumbar burst fracture patients during the perioperative period was 303.5 ml, accounting for 67.5% of TBL. In literature, it is very rarely reported. In this study, we identified independent risk factors associated with positive HBL as follows: age, duration from admission to surgery, duration of operation, P_1_%, preoperative total cholesterol, and preoperative fibrinogen.

Based on our research data and literature reports, we presume that the reasons may be as follows. Bone marrow hematopoiesis and the ability to store red blood cells in the body decline with increasing age and poor compensatory ability for blood loss anemia, resulting in a prolonged postoperative hematocrit in a low state, ultimately leading to high hidden blood loss calculated by gross formula [24, 25]. In a subset of patients with COPD as well as a long history of smoking, there is an increased amount of superoxide anion in the body, decreased capillary elasticity, and increased fragility. Meanwhile, because thoracolumbar burst fractures are high-energy injuries, the stress and inflammatory response induced by trauma and anesthesia can lead to increased peripheral capillary permeability and damage to the vascular endothelium [24, 26]. These insults result in diminished regulatory capacity of the peripheral capillary bed, allowing increased blood loss and impaired blood return.

Surgical trauma may cause a stress response, which stimulates the synthesis of unsaturated fatty acids such as cholesterol and triacylglycerol in the blood. In our work, we observed that the concentrations of cholesterol and triacylglycerol were higher in the HBL (+) group compared with the HBL (−) group. Consequently, cholesterol was indicated as a risk factor for HBL (+) in multivariate logistics regression analysis. However, to the best of our knowledge, there is very little literature that explores this association. Based on previous literature, we suggest that non-esterified fatty acids may have played an important role in oxidative stress [27, 28]. Unsaturated fatty acids stimulate reactive oxygen species (ROS) activation in neutrophils [29]. Subsequently, activated ROS attacks erythrocyte membrane proteins via nicotinamide adenine dinucleotide oxidase, causing membrane perforations and thus disruption of the plasma membrane and having an indirect hemolytic effect [29].

An important concern in the treatment of thoracolumbar burst fracture is timing [30–32]. Generally, the timing of the operation depends on the severity of the nerve injury. At present, there is a preliminary consensus on the timing of surgery for incomplete nerve injury. Compared with complete nerve injury, patients with incomplete nerve injury can benefit more from early emergency decompression [22, 32]. However, the definition of an early surgical time window demands serious consideration.

Most previous studies believed that the early surgical time window was 24–72h after injury, but some scholars also believed that the early operation time should be within 12–24h after injury [30, 32]. Based on published literature and our experience, we defined the early time window as within 48 h after injury. Multivariate logistic regression analysis identified surgical time window < 48h as an independent risk factor for positive HBL. In a previous retrospective study, patients were divided into 3 groups according to the timing of surgery for patients with thoracolumbar burst fractures. The timing of patients undergoing surgery in different groups was categorized as 12–24 h post-injury, 3–5 days post injury, and 7–10 days post-injury. One interesting finding was that the proportion of HBL to TBL in these 3 groups was 52.43%, 45.10%, and 49.41%, respectively. Traumatic stress may have played an important role in it. Traumatic stress via stimulation of tumor necrosis factor-α (TNF-α), interleukins (IL), and other cytokines are synthesized to inhibit erythroid formation. At the same time, bone fractures lead to disruption of the local microcirculation and local tissue hypoxia, thereby causing activation of the anaerobic glycolytic pathway and an increase in lactate concentration [33]. These all stay, causing telangiectasia, leakage of tissue fluid, and inducing a fall in effective circulating blood volume. When patients undergo emergency surgery, it is often difficult to correct the homeostasis resulting from trauma in a timely manner. Therefore, we would have resulted in an enlarged HBL when calculated using the Gross formula.

Interestingly, our results demonstrated that P_1_% was identified as a risk factor for HBL, which could presumably be ascribed to blood infiltration into the tissue compartments. Namely, the higher P_1_%, the more penetrable tissue compartments could be formed. It means that the more the vertebral height is restored, the bigger the “cavity” will be [7]. Meanwhile, with increased vertebral height restoration, there will be a larger fracture gap around the vertebral walls, which may lead to more infiltration of blood into the tissue compartments. Another noteworthy finding in our study was that fibrinogen was identified as a risk factor for HBL. Fibrinogen is an inflammatory protein that gets converted to fibrin in the presence of thrombin and directly influences platelet adhesion and activation. It was previously shown that levels were negatively correlated with HBL, which was similar to our findings [8, 34].

Here, we developed a novel predictive nomogram for predicting the risk of positive HBL in single-level TBF patients during the perioperative period. Our nomogram can demonstrate the key parameters graphically and can individually evaluate the incidence of risk of positive HBL. We believe it can contribute to clinical decision-making and doctor-patient communication. More importantly, we can identify patients with a high risk of positive for HBL. Additionally, the nomogram may provide opportunities that might lead to improvements in perioperative management.

There were a few limitations to our study that should not be ignored. First, due to the retrospective and observational nature of the study, the results are subject to limitations. Second, there was a lack of external validation for our proposed model, especially in other regions and countries. Third, specific data could not be obtained from the medical records or was missing. Finally, only patients with single-level TBF were enrolled. Patients with multi-level TBF need to be further studied in the future. Therefore, in future studies, our team should collaborate with the remaining medical centers and provide more data for more in-depth evaluation and validation of prediction models.

## Conclusions

In summary, by using a single center data in our hospital, we investigated the risk factors for HBL. Consequently, we established an individualized nomogram prediction model for positive HBL. Our nomogram is with satisfied performance in the evaluation of the risk of positive HBL, which may help clinical decision making. However, external validation will be needed in the future.

## Supplementary Information

**Additional file 1: Figure S1.** The receiver operating characteristic (ROC) analysis was used to determine the optimal cut-off values for continuous variables.

## Data Availability

The data set supporting the conclusion of this article is available on request to the corresponding author.

## Abbreviations

HBL: Hidden blood loss
TBF: Thoracolumbar burst fracture
AUC: Area under the receiver operating characteristic curves
DCA: Decision curve analysis
PLDF: Posterior lumbar decompression and fusion
MIS-TLIF: Minimally invasive transforaminal interbody fusion
PLIF: Posterior lumbar internal fixation
CSF: Cerebrospinal fluid leakage
BMI: Body mass index
COPD: Chronic obstructive pulmonary disease
JOA: Japanese Orthopaedic Association
VAS: Visual analogue score
SF-36: 36-Item Short Form Health Survey
P_1_%: Percentages of vertebral height restoration
P_2_%: Percentages of vertebral height loss
Hct: Hematocrit
Hb: Hemoglobin
ALB: Albumin
PT: Prothrombin time
APTT: Activated partial thromboplastin time
TLICS: Thoracolumbar Injury Classification and Severity Score
ROS: Reactive oxygen species
TNF-α: Tumor necrosis factor-α
IL: Interleukins
