# Supplementary material for: Development and validation of a nomogram for prediction of the risk of positive hidden blood loss in the perioperative period of single-level thoracolumbar burst fracture
Source: J Orthop Surg Res. 2021 Sep 15;16:560. doi: 10.1186/s13018-021-02699-6 (PMC8442389; doi:10.1186/s13018-021-02699-6)
Supplement: Supplementary file 1 — Additional file 1: Figure S1. The receiver operating characteristic (ROC) analysis was used to determine the optimal cut-off values for continuous variables. [file 13018_2021_2699_MOESM1_ESM.pdf]

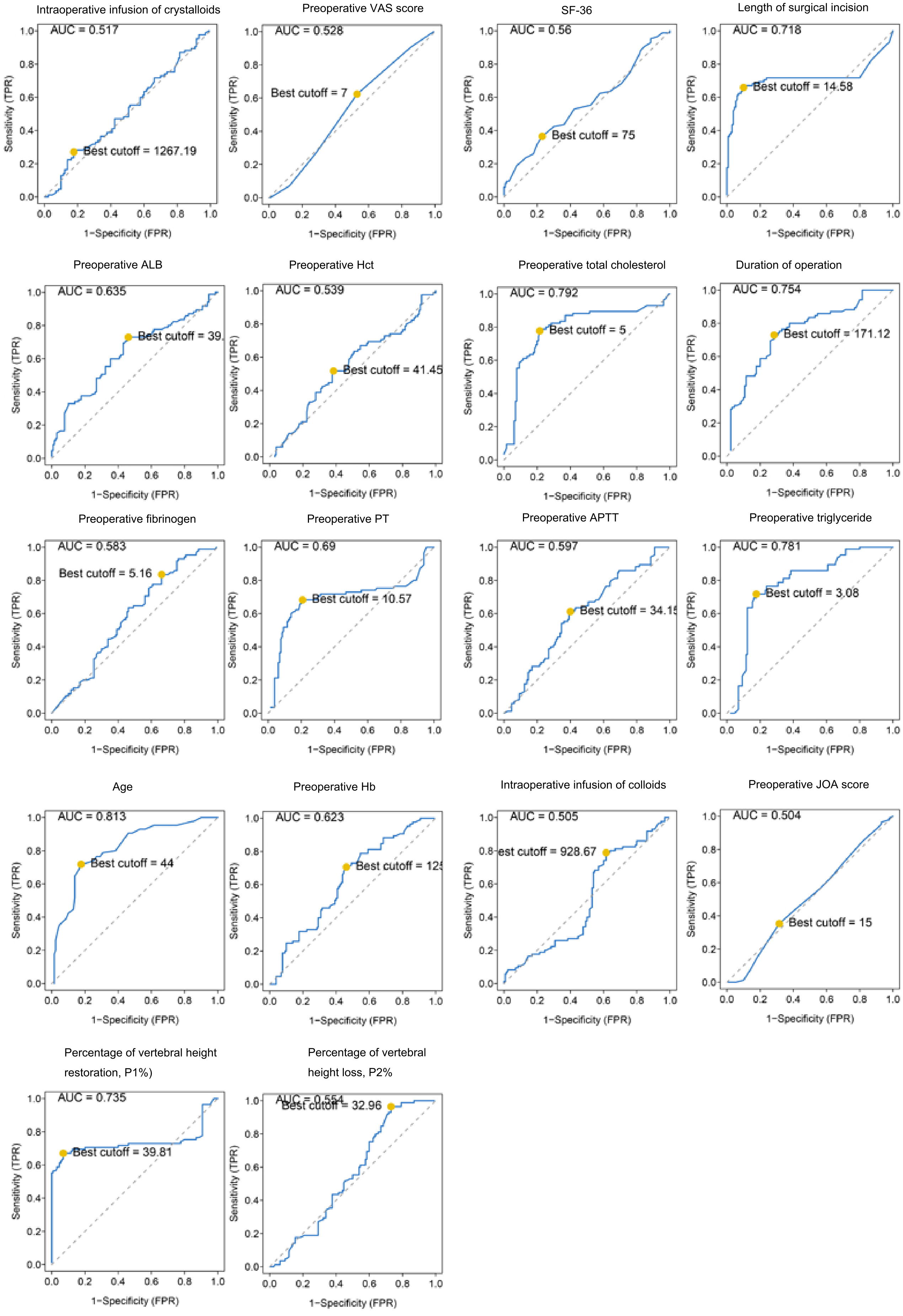

Intraoperative infusion of crystalloids
AUC = 0.517
Best cutoff = 1267.19
Preoperative VAS score
AUC = 0.528
Best cutoff = 7
SF-36
AUC = 0.56
Best cutoff = 75
Length of surgical incision
AUC = 0.718
Best cutoff = 14.58
Preoperative ALB
AUC = 0.635
Best cutoff = 39.
Preoperative Hct
AUC = 0.539
Best cutoff = 41.45
Preoperative total cholesterol
AUC = 0.792
Best cutoff = 5
Duration of operation
AUC = 0.754
Best cutoff = 171.12
Preoperative fibrinogen
AUC = 0.583
Best cutoff = 5.16
Preoperative PT
AUC = 0.69
Best cutoff = 10.57
Preoperative APTT
AUC = 0.597
Best cutoff = 34.15
Preoperative triglyceride
AUC = 0.781
Best cutoff = 3.08
Age
AUC = 0.813
Best cutoff = 44
Preoperative Hb
AUC = 0.623
Best cutoff = 125
Intraoperative infusion of colloids
AUC = 0.505
est cutoff = 928.67
Preoperative JOA score
AUC = 0.504
Best cutoff = 15
Percentage of vertebral height restoration, P1%)
AUC = 0.735
Best cutoff = 39.81
Percentage of vertebral height loss, P2%
AUC = 0.554
Best cutoff = 32.96
Sensitivity (TPR)
1−Specificity (FPR)
